# Clinical features of Crohn disease concomitant with ankylosing spondylitis: A preliminary single-center study

**DOI:** 10.1097/MD.0000000000004267

**Published:** 2016-07-18

**Authors:** Song Liu, Jie Ding, Meng Wang, Wanqing Zhou, Min Feng, Wenxian Guan

**Affiliations:** a Department of General Surgery; b Department of Laboratory Medicine,Nanjing Drum Tower Hospital, the Affiliated Hospital of Nanjing University Medical School, Nanjing, China.

**Keywords:** ankylosing spondylitis, Crohn's disease, epidemiology, feature, inflammatory bowel disease, outcome

## Abstract

Extraintestinal manifestations (EIMs) cause increased morbidity and decreased quality of life in Crohn disease (CD). Ankylosing spondylitis (AS) belongs to EIMs. Very little is known on the clinical features of CD concomitant with AS. This study is to investigate the clinical features of CD patients with AS.

We retrospectively collected all CD patients with AS in our hospital, and established a comparison group (CD without AS) with age, sex, and duration of Crohn disease matched. Clinical information was retrieved for comparison.

Eight CD + AS patients were identified from 195 CD patients. Sixteen CD patients were randomly selected into comparison group. All CD + AS patients were male, HLA-B27 (+), and rheumatoid factor (−) with an average age of 40.8 ± 4.52 years. Significant correlation between disease activity of CD and AS was revealed (*r* = 0.857, *P* = 0.011). Significant correlation between disease activity of CD and functional limitation associated with AS was identified (*r* = 0.881, *P* < 0.01). C-reactive protein (CRP), erythrocyte sedimentation rate (ESR), and globulin were positively correlated to Crohn disease activity index (CDAI), Bath AS disease activity index, and Bath AS functional index(BASFI) scores (*r* = 0.73–0.93, *P* < 0.05). Albumin was negatively associated with CDAI and BASFI (*r* = −0.73 to −0.91, *P* < 0.05). The ratio of albumin to globulin (Alb/Glo) was significantly related to all 3 scores (*r* = −0.81 to −0.91, *P* < 0.05).

Male predominance with a 4.12% concomitant incidence of AS is observed in CD patients. Disease activity of CD correlates with disease activity of AS and functional limitation caused by AS. CRP, ESR, and Alb/Glo may serve as biomarkers for disease activity and functional limitation in CD patients concomitant with AS, although future studies are expected.

## 1 Introduction

Crohn disease (CD) belongs to inflammatory bowel disease with an increasing prevalence across the world, including China.^[1]^ Besides gastrointestinal lesions such as ulcer, obstruction, and even penetration, a variety of extraintestinal manifestations (EIMs) can result from CD as well. CD patients with EIMs have been associated with increased morbidity and worse quality of life compared to their counterparts without EIMs.^[2,3]^ Therefore, understanding the clinical characteristics and designing management strategy toward EIMs in CD becomes a major challenge for clinicians.

Spondyloarthropathies (SpAs), as one of EIMs in CD, represent a group of distinct disorders with axial symptoms, peripheral arthritis, dactylitis, and enthesopathy. Ankylosing spondylitis (AS) is the most typical presentation of SpA, which mainly affects axial skeleton, leading to sacroiliitis, spondyloarthritis, spondylitis, and spondylodiscitis.^[4]^ Previous studies reported that the concurrent incidence of AS in CD was 1% to 5% in United States, whereas 2% to 15% in Europe.^[2,5]^ Comparison of clinical features and concomitant EIMs of CD between different races (e.g., African-American and white) is being undertaken to better instruct future genetic studies.^[6]^ However, data regarding epidemiology and clinical features of CD concomitant with AS in Chinese population are still very limited, which hamper therapy tailoring and genotype–phenotype investigations.

In the present study, we will collect all CD patients with AS registered in our center for prevalence calculation. Furthermore, we will compare their clinical features to CD patients without AS, and investigate the interaction between CD and AS by analyzing the correlation of disease activity and functional limitation associated with two diseases. We will also propose the surveillance and predictive value of certain serum biomarkers by analyzing theirresponses to fluctuating activity of CD and AS in Chinese patients.

## 2 Methods

### 2.1 Patients

We retrospectively scanned all patients with CD registered in the Department of General Surgery or Gastroinestinal Unit at our hospital between August 2011 and September 2015. The inclusion criteria included: a definitive diagnosis of CD (the diagnostic criteria of CD were described below); the reason of their first visit to our hospital is CD-associated symptoms or CD-associated complications; the absence of previous history of surgery or medication before the first visit to our hospital; for patients who re-visited our hospital during the study period, the data were only collected during their first visit.

A total of 194 qualified patients were yielded, among which 8 patients concomitant with AS were identified and subsequently enrolled into CD + AS group. To establish a comparison group, we randomly selected 16 patients with CD (2:1 ratio) from the Medical Record Database of our hospital (CD group). Age, sex, and duration of CD were matched between CD and CD + AS group. Propensity score was calculated to adjust confounding elements between experimental group and comparison group.^[7]^

The diagnostic criteria of CD included the following aspects^[8]^: a history of abdominal pain, vomiting, diarrhea, weight loss, or rectal bleeding; radiologic findings of stricture or fistula formation, mucosal cobble stoning, or deep ulceration; endoscopic appearances of cobble stoning, linear ulceration, or skip lesions; and pathologic confirmations of transmural inflammation or noncaseating epithelioid granulomas.

The diagnostic criteria of AS were in accordance to the modified New York criteria that included both clinical and radiological aspects.^[9]^ Clinical criteria included: low back pain and stiffness for >3 months, which improve with exercise, not relieved by rest; limitation of motion of the lumbar spine in both the sagittal and frontal planes; and restriction of chest expansion relative to normal values corrected for age and sex. Radiological criterion was bilateral sacroiliitis grade ≥2 or unilateral sacroiliitis grade 3 to 4. A definite AS was yielded by the radiological criterion and at least 1 clinical criterion.

This study has been approved by the Ethics Committee of Nanjing Drum Tower Hospital. As a retrospective study, an informed consent from enrolled individuals was not required by the Ethics Committee.

### 2.2 Data collection

For each patient, the sociodemographic information, disease-related characteristics, and laboratory results before treatment were recorded at the time of their first visit. The natural history, surgery, and medication data as well as clinical prognosis were retrieved from the electronic database for subsequent analysis.

The Montreal classification of CD was adopted in the present study.^[10]^ The disease location was classified into L1 (terminal ileum), L2 (colon), L3 (ileocolon), and L4 (upper gastrointestinal tract). The disease behavior was classified into B1 (inflammatory), B2 (stricturing), B3 (penetrating), and P (perianal lesion).

Crohn disease activity index (CDAI) score representing disease activity of CD, Bath AS disease activity index (BASDAI) score representing disease activity of AS, and Bath AS functional index (BASFI) score representing functional limitation of associated with AS were all collected at the time of their first visit to our hospital. The scoring criteria of above indexes were in accordance to previous publication by respective academic society.^[11–13]^

### 2.3 Statistics

Continuous variables were expressed as mean ± SE (standard error) and compared with unpaired student *t* test in addition to Welch correction when equal variance was not assumed. Discrete variables were presented as frequencies (percentages), and compared with *χ*^2^ test or Fisher exact test. All calculated data are presented with 3 significant figures. Correlation between biochemical parameters and disease-related scores was investigated with nonparametric Spearman correlation analysis. All statistical analyses in this study were performed using GraphPad Prism Software (version 5.01;GraphPad, San Diego, CA).

## 3 Results

**Figure 1:**
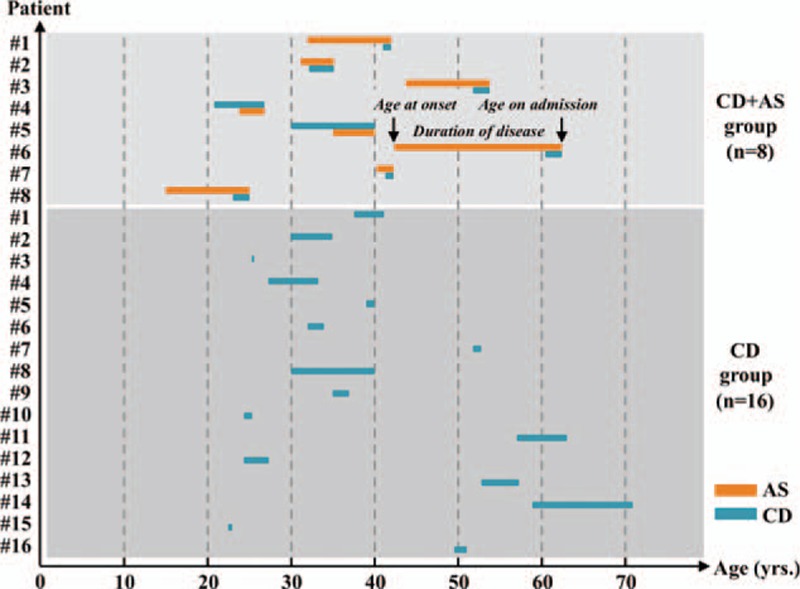
Course of disease in all enrolled patients. The starting and ending points of each rectangular box represent the age at disease onset and age on admission of each patient, respectively. The length of box illustrates the duration of disease. Orange and blue boxes correspond to AS and CD, respectively. The overlap of orange and blue boxes indicates the concomitance of 2 diseases in the same patient. AS = ankylosing spondylitis, CD = Crohn disease.

The overall incidence of AS in patients with CD was 4.12%. All 8 patients in CD + AS group were male with an average age of 40.8 ± 4.52 years. We therefore randomly selected 16 male patients with a matching age of 40.6 ± 3.64 years for comparison (*P* = 0.984). The duration of CD in 2 groups was similar (3.69 ± 0.863 vs 3.33 ± 1.11 years, *P* = 0.810). Notably, the course of AS was significantly longer than that of CD (7.95 ± 2.10 vs 3.33 ± 1.11 years, *P* = 0.0308) in CD + AS group, indicating that AS was likely to occur earlier than CD. Indeed, Figure 1 demonstrated that in 75% patients, the symptom of AS appeared before the symptom of CD. None of enrolled patients declared a family history of CD. In terms of family history of AS, 25% patients in CD + AS group claimed positive family history of AS (including one case of father and the other case of elder brother), while none of patients in CD group had a positive family history of AS (*P* = 0.101) (Table 1).

**Table 1:**
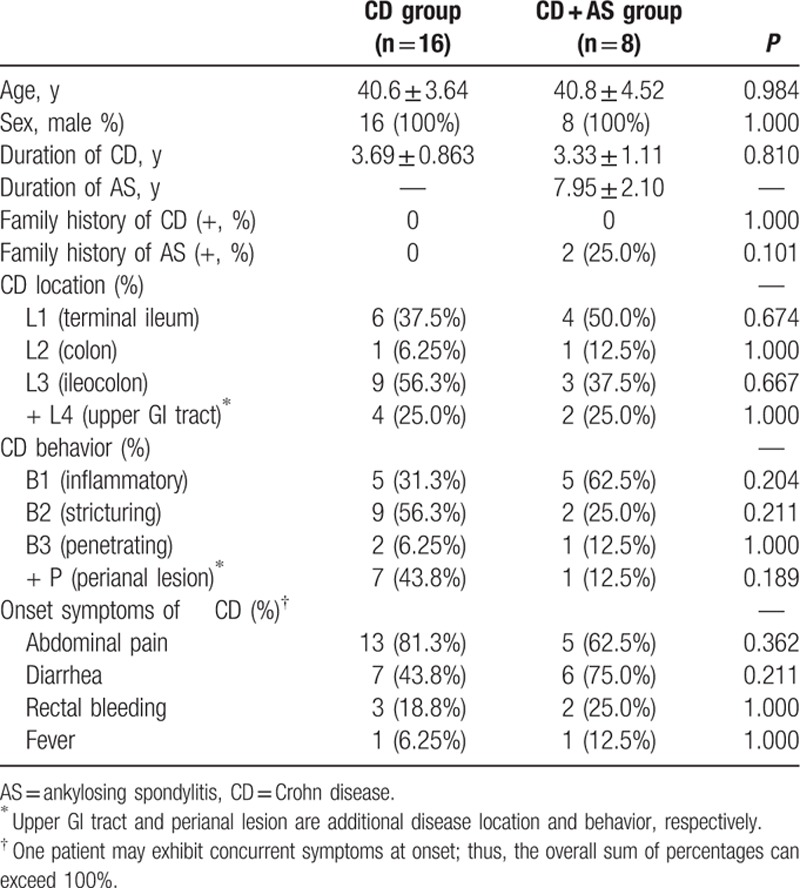
Demographics and disease-related characteristics of enrolled patients.

The Montreal classification revealed that the distributions of CD location and behavior were similar between 2 groups, although L1 (terminal ileum) (50.0%) and B1 (inflammatory) (62.5%) were mostly common in CD + AS group, whereas L3 (ileocolon) (56.3%) and B2 (stricturing) (56.3%) were mostly frequent in CD group. Same frequency (25.0%) of upper gastrointestinal tract involvement was observed between 2 groups, although more involvement of perianal lesion in CD group (43.8% vs 12.5%, *P* = 0.189) was observed. The most frequent symptoms at onset of CD included abdominal pain (81.3% in CD group, 62.5% in CD + AS group, *P* = 0.362), diarrhea (43.8%in CD group, 75.0% in CD + AS group, *P* = 0.211) and rectal bleeding (18.8% in CD group, 25.0% in CD + AS group, *P* = 1.000). Statistical difference in the onset symptoms was not found between 2 groups. More than half of enrolled patients exhibited concurrent symptoms at onset (Table 1).

Next, we investigated the cross-sectional laboratory test results on admission. All patients in CD + AS group were HLA-B27 (+) and rheumatoid factor (RF) (−), indicating an indispensable correlation with HLA-B27 and an independent correlation with RF. HLA-B27 result of candidates in CD group was unavailable. Four patients in CD group who were suspiciousof concomitant RA on admission received RF examination for differentiation diagnosis. None of them were RF (+) (Table 2).

**Table 2:**
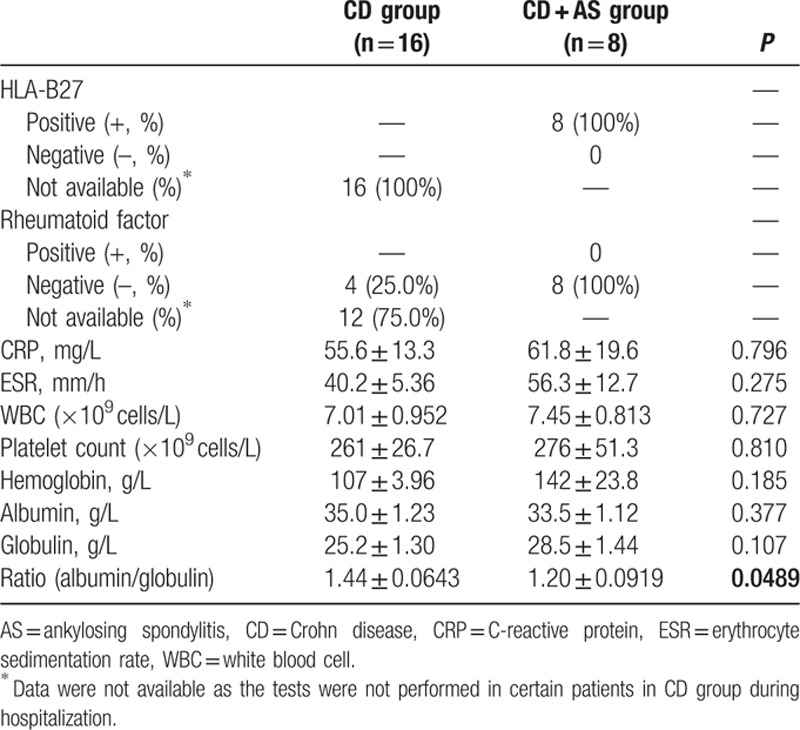
Comparison of laboratory data between CD and CD + AS group.

Serum inflammatory biomarkers, including C-reactive protein (CRP) and erythrocyte sedimentation rate (ESR), were similar between 2 groups, although the elevation of both CRP and ESR was detected, suggesting an activated immune status in vivo. Level of white blood cells (WBC) and platelet was within the normal range in the absence of statistical difference between 2 groups. Nutrition-related biomarkers, including hemoglobin, albumin, and globulin were all similar between 2 groups. However, the ratio of albumin to globulin was significantly higher in CD group compared to CD + AS group (1.44 vs 1.20, *P* = 0.0489) (Table 2).

We further compared the medication and surgery history between groups. As shown in Table 3, 5-amino saliciylic acid (5-ASA) including sulfasalazine and mesalazine was the fundamental drug for the treatment of CD, which was prescribed in 87.5% of CD patients. Corticosteroid and immunosuppressants (e.g., azathioprine, cyclophosphamide, and methotrexate) were also common drugs in both groups. Monoclonal tumor necrosis factor (TNF)-α antibody (infliximab) for CD was used in 18.8% and 25.0% of patients in CD and CD + AS group, respectively. Another TNF-targeting antibody (Etanercept) was used in 37.5% of patients for AS management. Antibiotics, mainly including metronidazole and tinidazole, were prescribed in 18.8% and 37.5% of patients as additional therapy in 2 groups, respectively. As the primary medication in the treatment of AS, NSAIDs (nonsteroidal antiinflammatory drugs) (mainly including celecoxib, etocoxib, loxoprofen and nimesulide) were applied in the majority (75.0%) of AS patients in our study. According to Table 3, 62.5% and 37.5% of patients in CD and CD + AS group underwent surgery (*P* = 0.391). Owing to higher prevalence of perianal lesions in CD group, 7 of 10 patients in contrast to 1 of 3 patients in 2 groups received perianal abscess drainage (*P* = 0.511). The remaining 3 patients in CD group received 2 partial intestine resection/anastomosis and 1 ileocecal resection for intestinal lesion removal. The remaining 1 patient in CD + AS group received partial intestine resection/anastomosis. Statistical difference in medication and surgery history was not obtained between 2 groups. The mean duration of follow-up period was 1.23 ± 0.21 years and 2.31 ± 0.52 years in 2 groups, respectively (*P* = 0.111). None of patients died from primary disease in the present study (Table 3).

**Table 3:**
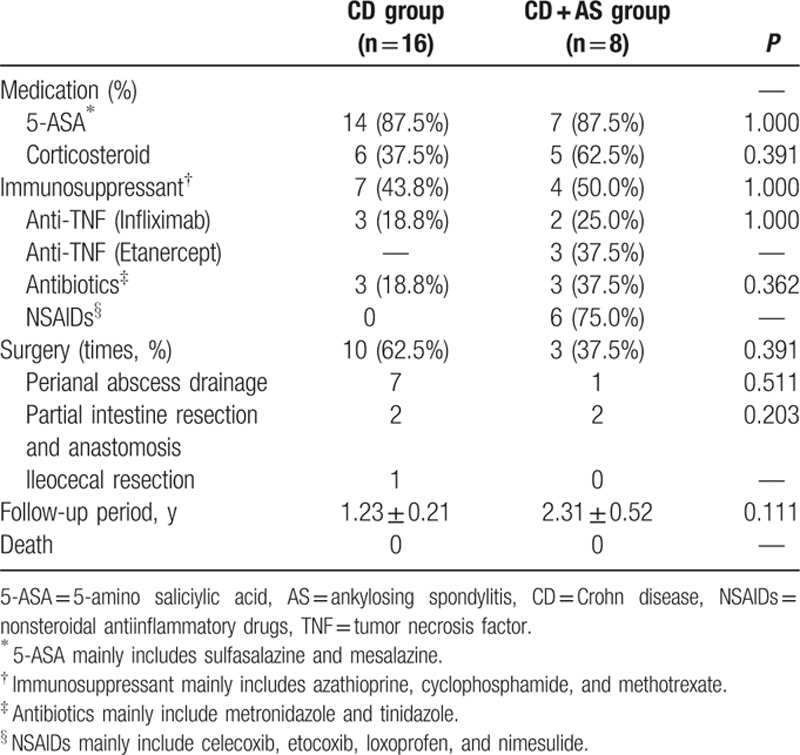
Clinical management and outcome between CD and CD + AS group.

**Figure 2:**
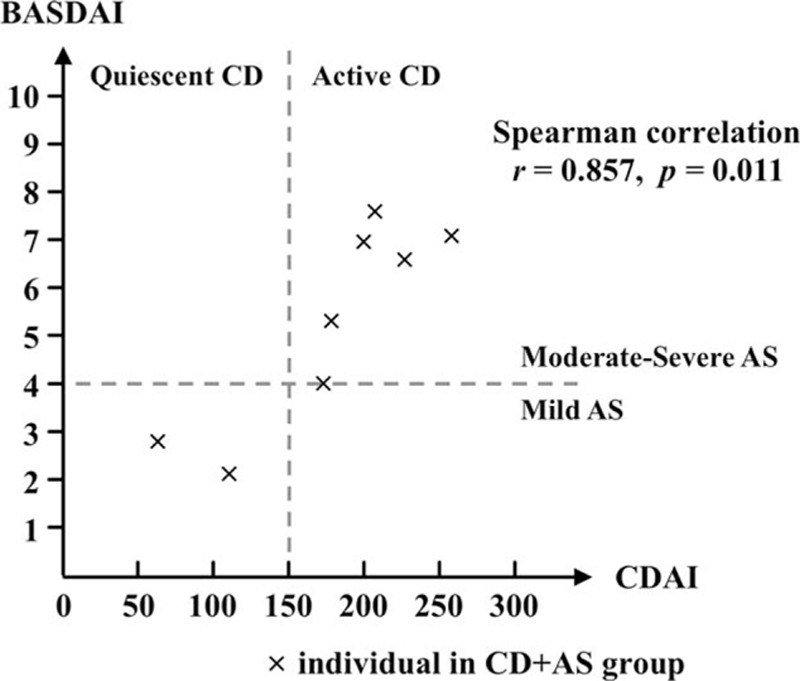
Correlation between disease activity of CD and AS. CDAI score and BASDAI score represent the activity of CD and AS, respectively. Each cross mark indicates a patient in CD + AS group. Active CD is defined as CDAI >150, and moderate to severe AS is defined as BASDAI >4. AS = ankylosing spondylitis, BASDAI = Bath AS disease activity index, CD = Crohn disease, CDAI = Crohn disease activity index.

**Figure 3:**
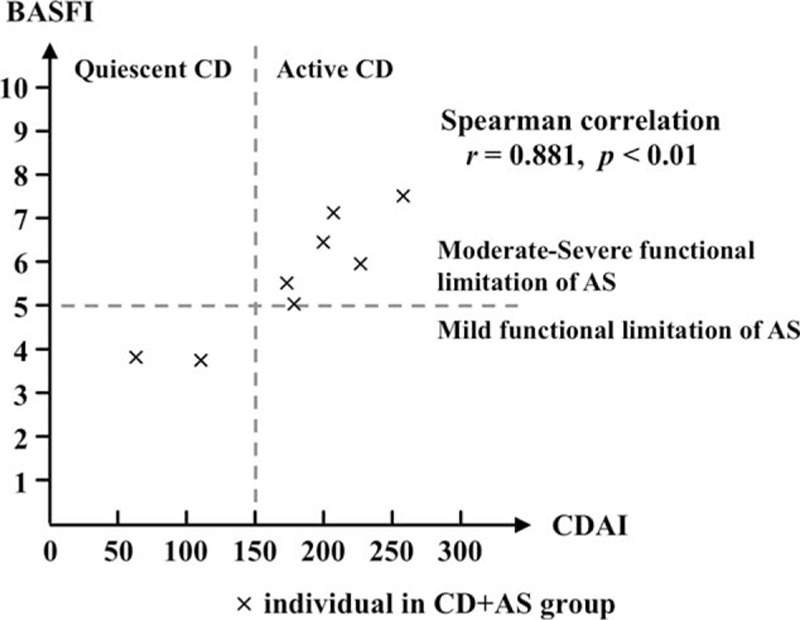
Correlation between disease activity of CD and functional limitation associated with AS. CDAI score represents the activity of CD, whereas BASFI score represents the functional limitation of AS. Each cross mark indicates a patient in CD + AS group. Active CD is defined as CDAI >150, and moderate-severe functional limitation in AS is defined as BASFI >5. AS = ankylosing spondylitis, BASFI = Bath AS functional index, CD = Crohn disease.

Next, we investigated the correlation between CD and AS. As shown in Figures 2 and 3, 6 patients with active CD (CDAI ≥150) show moderate to severe activity of AS (BASDAI ≥4) and moderate to severe functional limitation related to AS (BASFI ≥5). An intimate correlation between disease activity of CD and AS was revealed by Spearman correlation analysis (*r* = 0.857, *P* = 0.011). Similarly, a significant correlation between disease activity of CD and functional limitation associated with AS was identified as well (*r* = 0.881, *P* < 0.01). These findings suggested an underlying interaction of pathophysiological events between CD and AS in host.

We further analyzed the production of representative serum biomarkers in response to heterogeneous disease activity of CD and AS. As shown in Table 4, both CRP and ESR positively correlated to CDAI, BASDAI, and BASFI, ranging from 0.73 to 0.93 (*P* < 0.05) assessed by Spearman analysis. Interestingly, albumin was discovered to be negatively associated with CDAI and BASFI with an *r* value ranging from −0.73 to −0.91 (*P* < 0.05), whereas globulin was positively correlated with all 3 scores, ranging from 0.85 to 0.91 (*P* < 0.05). Furthermore, the ratio of albumin to globulin was significantly related to CDAI, BASDAI, and BASFI with an *r* value ranging from −0.81 to −0.91 (*P* < 0.05). In contrast, WBC, platelet, and hemoglobin were found irrelevant to any scoring index (Table 4).

**Table 4:**
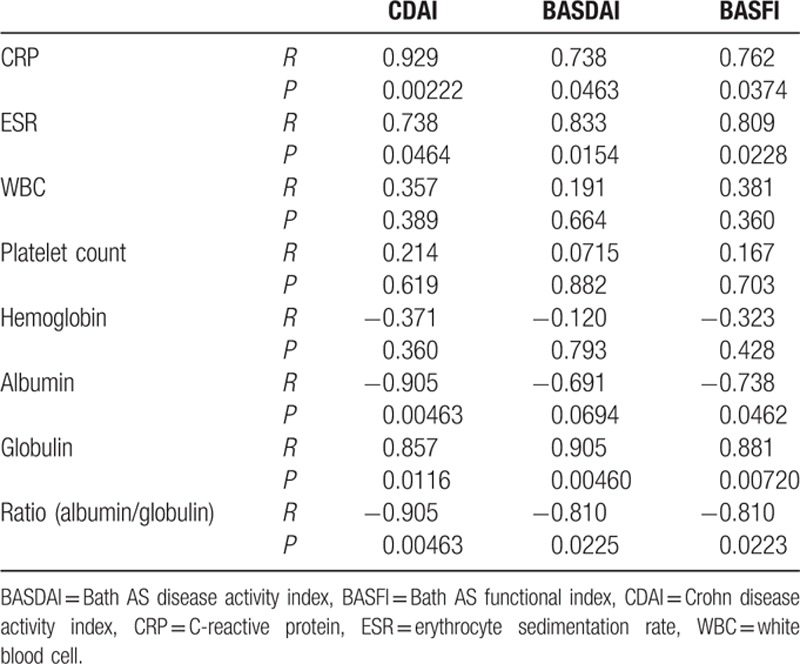
Correlation between biochemical index and disease-related scores.

## 4 Discussion

In current study, we retrospectively collected 8 patients diagnosed as Crohn's disease concurrent with AS, and analyzed their clinical features compared to 16 patients diagnosed as Crohn's disease without AS.

Herein, we summarized several important findings in our study: the concomitant incidence of AS in Chinese CD patients was 4.12%, which is the first report regarding the concomitant incidence of AS in CD patients from Mainland China; an overwhelming male predominance was observed in patients with CD + AS, indicating that male patients exposed to higher incidence of CD complicated with AS; the symptoms of AS appeared earlier than CD in majority of patients, suggesting that CD was presumptively secondary to AS; a trend of less perianal involvement was discovered in patients with CD + AS compared to patients with CD, implying a possible role of AS in preventing from perianal lesion in CD patients, although future mechanistic studies are expected for elucidation; a close correlation between disease activity of CD and AS was identified, suggesting intimate pathophysiological interactions between CD and AS in host; a series of serum parameters (including CRP, ESR, albumin, globulin, and the ratio of albumin to globulin) significantly correlated to CDAI, BASDAI, and BASFI scores, indicating that they were able to represent the disease activity and functional limitation associated with CD and AS, and therefore might become useful biomarkers in the surveillance of disease activity and the evaluation of therapy efficacy.

We firstly compared the concurrent prevalence and sex predominance of AS in CD between Chinese and western population. Leong et al^[14]^ reported that the concomitant prevalence of AS in CD patients was 9% according to their single-center study in Hong Kong. As Hong Kong cannot geographically represent Mainland China, their data were unable to represent the entire population of China. Beslek et al^[15]^ reported that the frequency of AS in Turkish CD patients was 14.3% with an equal sex distribution, whereas Isene et al^[2]^ reported a 1.6% incidence of AS in CD patients from 9 centers in 7 European countries. Vavricka et al^[16]^ reported that the incidence of AS in CD was 5.7% in a large Swiss inflammatory bowel disease (IBD) cohort. Bernstein et al compared the incidence of AS in female and male CD patients in the United States, and found that AS was much more common in men (2.7%) instead of women (0.7%). Moreover, they found that male CD patients were at higher risk of for concomitance during 10-year follow-up period (odds ratio [OR] = 17.7 in men vs OR = 3.9 in women),^[17]^ which is in accordance to our findings. Shivashankar et al^[5]^ reported that the cumulative incidence of AS in the United States was 0, 0.5%, and 0.5% within 10, 20, and 30 years after CD diagnosis, respectively. Sofia et al^[6]^ compared the AS involvement between African-American and white CD patients, and concluded that the concomitant incidence of AS was similar between 2 races (2.8% vs 2.9%). Cohen et al^[18]^ revealed that the concomitant risk of AS among patients with CD was 13.6 to 16.3 in US national cohort, but they did not analyze the sequential onset of AS and CD. Interestingly, Haraoui et al^[19]^ described a series of patients with AS developed CD following the treatment of Etanercept. They presumed that Etanercept could unmask or reactive gastrointestinal symptoms. In current setting, 6 of 8 patients developed CD after AS, among which 3 patients received Etanercept. We proposed that Etanercept would partly contribute to the late appearance of CD in AS patients because of unique but unidentified therapeutic mechanism of Etanercept compared to other anti-TNF drugs (e.g*.,* Infliximab).

Obviously, the prevalence of AS in CD varies dramatically between ethnics, areas, and studies. Consistent result cannot be achieved yet. Moreover, data from Asia-Pacific region are still unavailable. We herein for the first time reported the concurrent incidence of AS, the male predominance pattern, and the appearing sequence of symptoms related to CD and AS in Mainland Chinese CD patients, which would contribute to future genotype–phenotype studies in this field.

Current literature has demonstrated that subclinical gut inflammation could be found in AS patients before they were diagnosed with CD. Earlier in 1957, Steinberg et al^[20]^ for the first time described the coexistence of AS and subclinical intestinal inflammatory lesions in 6 patients. Later in 1985, Smith et al^[21]^ discovered significantly increased bowel permeability in patients with AS compared to controls, suggesting an immunological interaction between AS and gut inflammation. Furthermore, Martinez-Gonzalez et al^[22]^ found that increased bowel permeability existed in both AS patients and their relatives, suggesting a genetic interaction between AS and gut. Recent studies focused on cytokine profile shared by AS and IBD,^[23]^ and initiated monoclonal antibody therapy simultaneously for both AS and IBD.^[24,25]^ All mentioned studies have consistently confirmed that AS could exert significant impact on gut immune responses, although underlying mechanism has not been fully revealed. In our study, biopsies of AS patients were unavailable. Patients who visit our hospital because of AS-associated reasons are excluded from this study, since one of the inclusion criteria is “the reason of their first visit to our hospital is CD-associated symptoms or CD-associated complications.” Nevertheless, further investigations toward the mechanism by which AS induces subclinical gastrointestinal inflammation and the associated clinical significance would be expected.

Perianal involvement happens frequently in Chinese patients with CD.^[26]^ In this study, we noticed that perianal lesion occurred less common in CD patients when concomitant with AS. We hypothesize that this phenomenon may be partly because of the limited sample size that brings in selection bias, and perhaps be partly attributed to the protective effect by concomitant AS to perianal lesion. This finding is awaiting further confirmation and elucidation toward underlying molecular basis.

Our data clearly demonstrated a synchronized pattern of disease flare or disease quiescence between AS and CD. Synchronization between disease activity of CD and functional limitation of AS was confirmed as well. These observations highlight the common pathophysiological events shared between AS and CD in vivo. Indeed, current literature has identified certain proinflammatory cytokines and signaling pathways that are shared in the pathogenesis of the 2 autoimmune disorders.^[27]^ Our findings remind clinicians to design management strategies for both AS and CD when either of them is active.

As a TNF-α blocker, Etanercept is effective and has been recommended for the treatment of AS. Although earlier clinical studies confirmed its efficacy in the treatment of CD,^[28]^ subsequent trials reached a consensus regarding the inefficiency and even adverse effect of Etanercept in the treatment of CD.^[29–31]^ Underlying mechanism could include the unresponsive T-lymphocytes in lamina propria to Etanercept.^[32]^ In our study, there were 3 patients in CD + AS group who received Etanercept for AS treatment before they were diagnosed as CD. The prescription of Etanercept was immediately terminated when CD was diagnosed. In these patients, it is possible that Etanercept accelerated the appearance of CD, in accordance to previous reports. Nevertheless, association between Etanercept prescription and onset of CD has not been fully demonstrated by current literature yet.

NSAIDs are the first-line drugs in the treatment of AS. However, NSAIDs have major gastrointestinal side effects and are not recommended for AS patients with previous or current IBD history according to recent evidences.^[33,34]^ In our study, the appearance of AS was earlier than the appearance of CD in majority of patients. These AS patients had been treated with NSAIDs before they were subsequently diagnosed as CD. Our results imply that NSAIDs could possibly induce the appearance of CD in AS patients, and remind physicians of being aware of NSAIDs usage in AS patients, especially when concomitant with suspicious subclinical gut inflammation.

Another finding in this study is the correlation between serum biomarkers and scoring index for disease activity and functional limitation associated to AS and CD. CRP and ESR are typical inflammatory biomarkers that have been widely used in CD^[35]^ and AS^[36,37]^ for the evaluation of disease activity and drug efficacy. Nutritional status represented by albumin has also been recommended in CD for the assessment of disease severity and the risk of postoperative complications.^[38,39]^ However, few studies have investigated the relationship between serum albumin and disease severity as well as functional limitation in AS patients. In the present study, we show that CRP and ESR could work as biomarkers for both CD and AS, and proposed the ratio of albumin to globulin as a major indicator of disease activity and functional limitation in patients with CD and AS. Future studies with large amount of patients would determine the cutoff value and corresponding diagnostic power (sensitivity and specificity) of above biomarkers in CD and AS.

RF (−) is not a mandatory item for the diagnosis of AS according to the modified New York criteria for AS diagnosis. However, considering that part of AS patients could concomitant with arthritis and associated joint pain, we routinely examined RF for patients with suspicious AS when they first visited our hospital to assist the differential diagnosis of AS from RA. In our study, all 8 AS patients were RF negative that could be expected.

Genetic analysis has been performing in an attempt to clarify the genetic background of the association between CD and AS. *HLA-B27* is the first and foremost gene. Although the importance of *HLA-B27* in conferring susceptibility to AS is well known, the role of *HLA-B27* in axial involvement in IBD patients is much less conclusive,^[40]^ as earlier studies reported diverse positive rates of *HLA-B27* (ranging from 25% to 75%) in CD patients.^[41,42]^ Notably, we herein reported that all patients with CD and AS presented positive for HLA-B27, which is quite inspiring. However, the unavailability of HLA-B27 result in comparison group owing to the nature of retrospective study makes it impossible to analyze the diagnostic value of HLA-B27 in CD patients suspicious of concomitant AS.

Danoy et al^[43]^ achieved interesting genetic findings recently. They discovered variants at *chr1q32* and *Stat3* that link AS and CD, and proposed that these risk variants might become the basis of shared pathogenesis. Nevertheless, future investigations are required to demonstrate corresponding molecular mechanism.

We are aware of our limitations in the present study. First, a potential risk of selection bias may exist because of the limited sample size from a single center, which may not fully represent the comprehensive relationship between CD and AS. To reduce potential selection bias, patients in comparison group were randomly yielded from the CD database. We chose 2:1 instead of 1:1 ratio as 2:1 ratio is frequently adopted in cross-sectional and case–control studies, which could enlarge the sample size and theoretically enhance the comparison precision. Based on the findings from this preliminary study, we are currently conducting a multiple-center study to recruit more qualified CD patients with extraintestinal manifestations. Second, this study lacks follow-up prospective data after receiving medical or surgical interventions, thus depriving the possibility to evaluate the value of serum biomarkers in the surveillance of treatment efficacy. Third, the follow-up period in this study is too short to unmask the difference of clinical prognosis between CD patients concomitant with AS or not. Nevertheless, data from randomized prospective study with large number of patients are still unavailable. We expect that our findings are useful for colleagues in the diagnosis and management of CD concurrent with AS.

## Acknowledgement

The authors thank Dr Daren Low (the Agency for Science, Technology and Research, Singapore) for his critical review and language polish of this article.
